# Restoring the Cell Cycle and Proliferation Competence in Terminally Differentiated Skeletal Muscle Myotubes

**DOI:** 10.3390/cells10102753

**Published:** 2021-10-14

**Authors:** Deborah Pajalunga, Marco Crescenzi

**Affiliations:** 1 Department of Oncology and Molecular Medicine, Italian National Institute of Health, 00161 Rome, Italy; deborah.pajalunga@iss.it; 2 Core Facilities, Italian National Institute of Health, 00161 Rome, Italy

**Keywords:** skeletal muscle, terminal differentiation, cell cycle, postmitotic state, regenerative medicine

## Abstract

Terminal differentiation is an ill-defined, insufficiently characterized, nonproliferation state. Although it has been classically deemed irreversible, it is now clear that at least several terminally differentiated (TD) cell types can be brought back into the cell cycle. We are striving to uncover the molecular bases of terminal differentiation, whose fundamental understanding is a goal in itself. In addition, the field has sought to acquire the ability to make TD cells proliferate. Attaining this end would probe the very molecular mechanisms we are trying to understand. Equally important, it would be invaluable in regenerative medicine, for tissues depending on TD cells and devoid of significant self-repair capabilities. The skeletal muscle has long been used as a model system to investigate the molecular foundations of terminal differentiation. Here, we summarize more than 50 years of studies in this field.

## 1. Introduction

TD cells are classically defined as specialized cells that have irreversibly lost their ability to proliferate (postmitotic state). This definition, however, is based on the indeterminate notion of “specialization” and on the absence of evidence of proliferation. Both pillars rest on soft ground. We do not know how to objectively measure specialization and what degree of this property, if any, entails terminal differentiation. As to the second pillar, the lack of evidence of proliferation cannot exclude that cells might divide under rare or special conditions. As a relevant example, adult cardiomyocytes, long considered postmitotic, are now established as being endowed with a limited but definite proliferative potential [1]. Indeed, there is ample evidence that at least the cell cycle—or even proliferation—can be reactivated in nearly any cell type, in natural or experimental conditions, and that the postmitotic state can no longer be considered irreversible.

However defined, TD cells, if belonging to tissues with limited or absent renewal, must live as long as their organism itself. This generates the evolutionary problem of ensuring their long-term survival through especially efficient maintenance and repair mechanisms. In addition, they represent a biological mystery, in that we have a limited understanding of the molecular mechanisms that trigger permanent exit from the cell cycle, of what locks the cells in the postmitotic state, and why such a state is so common in mammals and other classes of vertebrates.

Some animals are able to perform amazing regeneration feats. The newt, a urodele amphibian, is among the best studied examples. Newts can regenerate virtually any part of their bodies, after injury. In these animals, the skeletal muscle, as well as many other tissues, can proliferate in response to damage and contribute to regenerate the missing parts. Hence, though quite similar to ours, the muscle of these animals can successfully reenter the cell cycle, divide, proliferate, and even redifferentiate into other lineages [2]. These notions allow the speculation that the postmitotic state might be reverted in favor of regeneration even in mammals.

Skeletal muscle myotubes are readily generated and easy to cultivate and manipulate in vitro, while the molecular details of their differentiation are understood in depth [3]. For these reasons, they constitute a time-honored model in studies of terminal differentiation. Indeed, mammalian skeletal muscle fibers are excellent examples of postmitotic cells, as under natural conditions they virtually never reenter the cell cycle. Scientists have generally investigated the postmitotic state of TD cells with two aims. On one side, they wish to understand the molecular mechanisms underpinning the decision to abandon proliferation and what makes this choice normally permanent. In doing so, they hope to penetrate the deep significance of the postmitotic state, and its evolutionary advantages and drawbacks. On the other side, they wish to discover how to induce TD cells to proliferate in a controlled, safe, and reversible fashion. Possessing such ability would offer great opportunities to regenerative medicine. It would be invaluable to replace cells lost to diseases or injuries of organs incapable of self-repair through parenchymal cell proliferation. Two general strategies can be envisioned. In ex vivo approaches, healthy TD cells, explanted from a damaged organ and expanded in vitro, would be then transplanted back to replace lost cells. A second possibility is exploiting similar methods for direct, in vivo tissue repair. Reactivation of the cell cycle in TD cells is to be regarded as an approach opposite but complementary to the mastery of stem cells for similar purposes.

Here, we review the history and the current state of the efforts to induce TD skeletal muscle cells to reenter the cell cycle and proliferate.

## 2. The Skeletal Muscle in Culture

In vivo, skeletal muscles develop through long and complex schemes [3]. In culture, investigations largely focus on a narrow window that includes adult satellite cells—the muscle-tissue reserve cells—and their differentiation into syncytial myotubes. Satellite cells, which are mostly quiescent in vivo, can be readily isolated and put into culture where, in the presence of growth factors [4,5], they proliferate and are termed myoblasts. The latter can be made to divide extensively and induced to differentiate in a growth factor-poor medium. Under these conditions, myoblasts permanently withdraw from the cell cycle (commitment stage), begin to express muscle-specific genes, and become mononuclear, TD myocytes. Finally, myocytes fuse with one another to generate multinucleated myotubes. These stages have been deeply studied on the molecular level [3,5,6].

Central to muscle development and differentiation are the four transcription factors (muscle regulatory factors, MRFs) of the MyoD family of bHLH (basic-helix-loop-helix) proteins [3,7]. The MRFs bind other bHLH proteins, such as ITF-2 and E12/E47, to generate DNA-binding heterodimers. The main function of the MRFs is to specify the skeletal muscle lineage (Myf5 and MyoD) or orchestrate differentiation (Myogenin), while MRF4 possesses aspects of both activities [8]. During differentiation, the MRFs exert their functions with the assistance of the Mef2 family of transcription factors. In addition to regulating transcription, at least some of the MRFs play critical chromatin remodeling roles. In particular, MyoD recruits a number of chromatin remodeling factors, including the SWI/SNF proteins BRG and BRM [9,10] and histone acetylases p300 and PCAF [11,12]. This function is essential to open chromatin and allow transcription factors to access muscle-specific regulatory regions, thus driving expression. 

**Figure 1:**
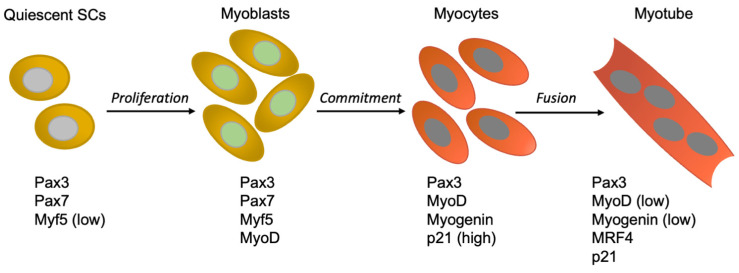
Expression of select genes during adult myogenic differentiation. Expression of the indicated genes in quiescent satellite cells (SCs), myoblasts, myocytes, and myotubes.

Although the MRFs are deployed in a fairly constant temporal order, they are interconnected and generally capable of regulating transcription of themselves and their family members [3]. Of the four MRFs, quiescent satellite cells express Myf5, along with the paired box transcription factors Pax3 and Pax7. The latter is restricted to satellite cells and thus constitutes a specific marker. When induced to proliferate, former satellite cells, now myoblasts, begin to express MyoD. Early after the induction of differentiation, myoblasts undergo commitment, which is normally a prerequisite for differentiation, cease expressing Pax7, and start transcribing Myogenin. Interestingly, at this stage, MyoD upregulates the cell cycle inhibitor p21, which plays a critical function in the maintenance of the postmitotic state (see later, The molecular cell cycle era). Eventually, myocytes fuse into myotubes, variably downregulate Myogenin and MyoD, and begin expressing MRF4 (Figure 1).

## 3. The Postmitotic State in Myotubes

**Figure 2:**
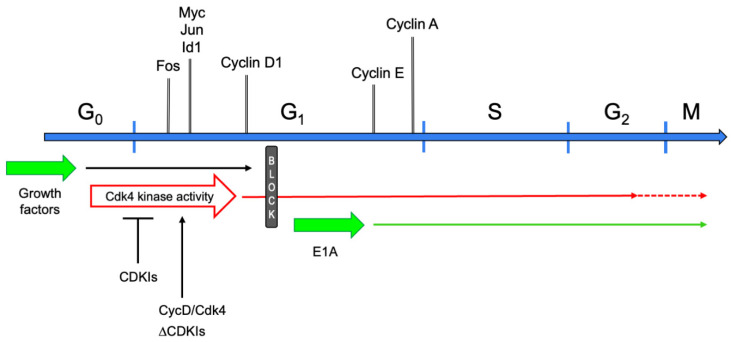
Schematic of the cell cycle in myotubes. Cell cycle phases are graphed as a linear succession. Above the cell cycle line, marker genes are shown at the approximate time point when they are first expressed or upregulated, when reentering the cell cycle from G_0_. Below the cell cycle line, the effects of several cell cycle-reactivating triggers are presented. Upon growth factor stimulation, TD myotubes exit G_0_ phase, enter G_1_, and progress up to the mid-G_1_ block, which they cannot pass. Expression of E1A makes myotubes jump from G_0_ to the G_1_-S boundary. They promptly induce expression of cyclin E and A, and progress into and beyond M phase. Cyclin D/Cdk4 overexpression (CycD/Cdk4) or CDKI depletion (ΔCDKIs) activates the Cdk4 kinase, allowing myotubes to reach S-G_2_ phase (CycD/Cdk4) or M phase (ΔCDKIs).

The postmitotic state has long been regarded as an attribute of TD cells that have ceased dividing and cannot be recalled into the cell cycle [13]. This definition suggested that such cells are permanently confined in G_0_ phase. Indeed, they do not synthesize DNA in response to any growth factors, nor to the forced expression of a variety of genes that are powerful mitogenic stimulators in non-TD cells [14]. This static view was initially challenged by the observation that myotubes stimulated with serum or individual growth factors re-express the early cell cycle gene c-Myc [15]. Subsequent studies investigated the control of the cell cycle in postmitotic myotubes in further detail. It was shown that these cells can be readily brought into G_1_ phase by growth factor stimulation [14]. In fact, the initial transcriptional responses to serum of reversibly quiescent myoblasts and myotubes are indistinguishable, comprising the expression of cell cycle genes such as Fos, Jun, Myc, Id1, and Cyclin D1. However, myotubes display no further response, beyond the expression of cyclin D1, leading to the postulation of a mid-G_1_ block that prevented these cells from progressing into S phase [14] (Figure 2). Interestingly, growth factor stimulation, though partially reactivating the cell cycle, did not suppress the expression of muscle-specific genes [14,15].

## 4. Early Attempts at Cell Cycle Reactivation

Initial attempts to reactivate the cell cycle in myotubes were carried out in the 1960s, using DNA tumor viruses. At the time, the ability of the polyoma and SV40 viruses (now both belonging to the Polyomaviridae family) to drive the cell cycle had been recently discovered and the investigations of their properties were at the cutting edge of cell replication studies. Primary skeletal muscle myoblasts—not myotubes—were infected with polyomavirus [16] or SV40 [16,17] and began expressing their respective large T antigen oncogene. Myotubes were obtained by inducing the myoblasts to differentiate promptly after infection, presumably before T antigens accumulated significantly. Such myotubes synthesized DNA and could even undergo mitosis [17]. These results indicated that DNA replication can be induced in TD myotubes. However, as only myoblasts can be infected by these viruses, some levels of viral proteins expressed early during differentiation might conceivably have prevented terminal exit from the cell cycle (commitment), impairing differentiation and making the myotubes capable of entering S phase [18].

Subsequent experiments were carried out with myoblasts inducibly expressing the temperature-sensitive A58 mutant of SV40 large T antigen [19]. The activation of large T in TD myotubes induced reentry into the cell cycle, DNA replication, and mitosis. However, as the reactivated myotubes underwent apoptosis, no long-term proliferation occurred. Altogether, these experiments established that, in TD muscle cells, the cell cycle can be fully reactivated, up to and including mitosis [17,20]. In myotubes, mitoses were always aberrant, displaying diverse combinations of chromosome fragmentation, missegregation, and confluence of multiple nuclei [21]. Notably, although resting myotubes are devoid of centrioles [22,23], these were usually present at mitosis. What was also frequent was the occurrence of cytokinesis, although variably resulting in mono- or polynucleated daughter cells [21].

In the late 1980s, another DNA tumor virus, human adenovirus, began to be engineered as a convenient vector for experimental and gene therapy purposes. Several years later, it was then exploited as a more flexible tool to probe and reactivate the cell cycle in TD cells. Adenoviruses carry several oncogenes and one of them, E1A, shares key properties with the SV40 large T antigen. Notably, though, adenoviruses have a wider host range than SV40 and, in addition, can infect nonproliferating cells, including myotubes. Infection of TD skeletal muscle cells with wild-type (wt) human adenovirus serotype 5 induced cell cycle reentry. Adenovirus mutants showed that, of the two main viral oncogenes, E1A and E1B, only the former was necessary to reactivate myotubes [24].

Mechanistically, E1A bypasses the mid-G_1_ block encountered by serum-stimulated myotubes, as shown by the fact that it induces neither the expression of cell cycle early genes, nor that of cyclin D1. Rather, E1A promptly switches on the E and A cyclins, near the G_1_/S boundary (Figure 2). Unfortunately, then, it provided no clue as to the nature of the mid-G_1_ block.

As with the SV40 large T antigen, E1A induced cytokinesis in reactivated myotubes. Cleavage furrows formed in an appreciable percentage of the reactivated cells, but usually not at all possible sites (i.e., between any two daughter nuclei) within a myotube. In the final stages of cell division, some of the midbodies contained DAPI-stained filaments of DNA, a condition that generally results in aborted cytokinesis [25]. Indeed, time-lapse recordings showed frequent such instances of regressing mitoses in myotubes [26,27]. Irrespective of whether cell division was successful or not, E1A-reactivated myotubes constantly displayed mitotic aberrations, ranging from relatively minor to gross [27].

Reactivation mediated by E1A is accompanied by at least the partial suppression of muscle-specific gene expression [28,29,30]. This is mediated by the repression of transcription of all the MRFs, except Myf-5 [31,32]. However, the trans-acting activity of all four MRFs, including Myf-5, is inhibited by E1A [31,32].

Notably, once myotubes are reactivated by E1A, they are capable of undergoing at least one more cell cycle, independent of the continuing activity of the oncogene. This conclusion was reached by activating for as little as six hours an estrogen-dependent, chimeric E1A-ER protein. Although, subsequently, E1A was demonstrably inactivated, the myotubes entered S phase only 18 h later and many of them underwent a second round of DNA replication, up to at least 30 h after estrogen withdrawal [27]. We speculate that perpetuation of the cell cycle in the absence of the reactivating stimulus was allowed by the de-differentiation brought about by E1A.

Importantly, all of the DNA tumor virus oncogenes named in this section share the ability to bind [33,34,35,36] and functionally inactivate [37,38] the retinoblastoma protein (pRb) tumor suppressor gene. This is critical, in view of the major roles played by pRb in establishing and maintaining the postmitotic state (see next section). However, pRb inactivation by a viral oncogene is not always sufficient to reactivate the cell cycle in myotubes. Indeed, the papillomavirus E7 oncogene, when expressed in myotubes, could not trigger DNA synthesis, despite reducing pRb levels, increasing Cyclin E expression, and eliciting E2F transcriptional activity [39].

## 5. The Molecular Cell Cycle Era

Beginning in the 1980s, our understanding of the cell cycle was revolutionized by the elucidation of its molecular mechanisms. It was natural to apply the recently acquired knowledge to identify cellular genes—as opposed to viral ones—capable of reactivating the cell cycle in TD cells.

The simultaneous overexpression of Cyclin D1 and the cell cycle kinase Cdk4 was found to attain this goal [40]. Recombinant adenoviruses carrying the two genes were used to bring myotubes efficiently into S phase (>70% of myotubes in a culture). The reactivated cells underwent DNA replication and entered G_2_ phase, where, in most cases, they remained arrested (Figure 2). Cell death followed thereafter. Interestingly, while quiescent cells can be brought into S phase by Cyclin D/Cdk4 or cyclin E/Cdk2 complexes [41,42], myotubes can be reactivated solely by expressing one of the D cyclins in conjunction with Cdk4, or its family member Cdk6. Other combinations of cyclins and cdks fail to reactivate TD skeletal muscle cells. In particular, the overexpression of Cyclin E and Cdk2 attains Cdk2 kinase activity levels comparable to those elicited by E1A, yet cannot trigger DNA replication in myotubes [40]. This specificity might owe to the ability of MyoD and Cdk4 to physically bind [43]. Indeed, it has been proposed that the two proteins oppose each other’s effect, determining at least in part whether a myoblast proliferates or undergoes differentiation [44].

Although myotube reactivation required both Cyclin D1 and Cdk4 to be expressed at levels far above physiological, the Cdk4 kinase activity was comparable to that measured in spontaneously proliferating myoblasts [40]. Altogether, these experiments prompted the conclusion that the block met by growth factor-stimulated myotubes in mid-G_1_ was due to their inability to activate the Cdk4 kinase (Figure 2). Indeed, reconstituting physiological levels of Cdk4 activity allowed myotubes to progress through the cell cycle [40].

The experiments just described raised the question as to why extreme overexpression of Cyclin D1 and Cdk4 proteins was needed to obtain normal levels of Cdk4 kinase activity. One plausible explanation was that high levels of one or more cdk inhibitors (CDKIs), expressed in TD cells, might prevent activation of the kinase. Indeed, the expression of large amounts of diverse CDKIs had been described in a variety of TD cells [45,46,47,48,49,50,51], including myotubes [45,52,53,54,55,56]. These studies established a strong correlation between the expression of one or more CDKIs and terminal differentiation. In addition, they showed that CDKIs are essential for the initiation of the postmitotic state in several TD cell types. A mechanistic role in maintaining the postmitotic state was also suggested, but not proven.

Proof of the causal role of CDKIs in preserving the postmitotic state was provided by suppressing p21 (Cdkn1a) in TD skeletal muscle cells [57] (Figure 2). Myotubes derived from the established myoblast cell line C2C12 [58,59] promptly reentered the cell cycle upon p21 depletion, even in the absence of exogenous growth factors. This finding required a mechanistic explanation: which cyclins and cdks triggered the myotube cell cycle, and why were growth factors dispensable? The solution was found in multiprotein complexes present in myotubes, containing Cyclin D3, Cdk4, and p21, along with other cell cycle regulators, including Cdk2, pRb, and PCNA [60]. Thus, it was hypothesized that p21 depletion allowed activation of preformed Cyclin D3/Cdk4 complexes. Such heterodimers would require growth factors neither to induce Cyclin D expression nor to promote cyclin/cdk assembly. Accordingly, while the depletion of p21 efficiently triggered cell cycle reentry, interfering with both p21 and Cyclin D3 abrogated cell cycle reentry. Similarly, expressing a Cdk4-dominant negative mutant prevented p21 suppression from inducing DNA synthesis [57]. These results also showed that, in p21-depleted myotubes, cell cycle reactivation is mediated exclusively by endogenous Cyclin D3/Cdk4 (or Cyclin D3/Cdk6) complexes.

Interestingly, while p21 suppression was sufficient to extensively trigger cell cycle reactivation in C2C12 myotubes, other CDKIs played a significant role in primary myotubes. In fact, only a small minority of the latter cells were reactivated by p21 depletion, but the suppression of p21 along with one or more other CDKIs (p18 (Cdkn2c), p27 (Cdkn1b), and p57 (Cdkn1c)) prompted progressively more cells to reenter the cell cycle. Nonetheless, p21 depletion was absolutely necessary to allow cell cycle reentry, suggesting that p21 is the primary inhibitor of the endogenous Cyclin D3/Cdk4 complexes and that other CDKIs partially substitute for it, following its removal. Surprisingly, p21 plays such a primary role, although, in C2C12 myotubes, p27 is 13-fold more abundant than p21 in molar terms. Even Cdk4-associated p27 is 6-fold more abundant than p21 is [57], confirming the specific role of p21 in the myotube model system.

**Figure 3:**
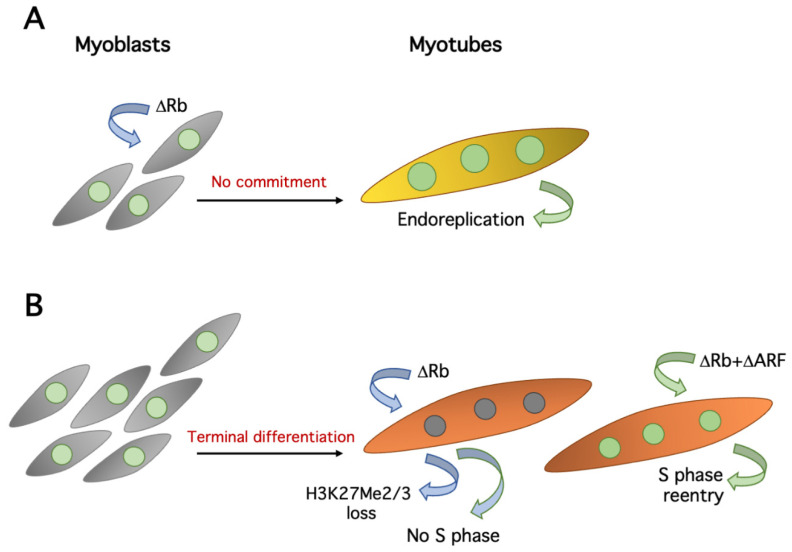
Effects of pRb suppression in primary myoblasts and myotubes. (**A**) Deletion of Rb in myoblasts allows defective myotube differentiation without the preceding commitment step, resulting in repeated cycles of endoreduplication (large nuclei). (**B**) Rb deletion alone causes the loss of H3K27Me2/3 on several cell cycle genes, but rarely triggers S phase. Complementary depletions of pRb and ARF initiate DNA replication.

Another important cell cycle regulator involved in muscle differentiation is pRb. In the early 1990s, it was suggested that pRb and MyoD interacted physically [61,62], as MyoD had been shown to inhibit proliferation [63,64,65]. Although a direct interaction was formally disproved [66], pRb does play a major role in muscle differentiation. Indeed, it was shown that, in the absence of pRb, myoblasts somehow differentiate, albeit with a reduced expression of “late” differentiation markers, such as the muscle-specific myosin heavy chain. However, they do not undergo commitment [61,67,68] (Figure 3A), normally a prerequisite for skeletal muscle differentiation [69]. In particular, it has been shown that pRb-deficient myotubes tend to undergo multiple rounds of DNA replication, in the absence of intervening mitoses (endoreduplication), both in vitro [68] and in vivo [70].

Once established that pRb is essential to initiate the postmitotic state in myotubes, it remained to be determined whether it is also necessary to maintain it. This was deemed plausible, as it had been already shown that both quiescence and senescence could be reverted by acutely ablating Rb [71]. However, using conditional Rb knockout mice, two reports showed that the removal of Rb from primary myotubes or muscle fibers impairs muscle-specific gene expression and activates the cell cycle machinery, but does not trigger DNA synthesis, in vitro or in vivo [72,73] (Figure 3B). In addition, it was shown that the whole pRb protein family, including p107 and p130, is dispensable for the maintenance of the postmitotic state of myotubes [73]. An ostensibly divergent study [74] reported that pRb depletion does reactivate the cell cycle in C2C12 myotubes. The simplest explanation for these apparently opposite results is that while the first two studies [72,73] were performed with primary muscle cells or in vivo, the more recent paper [74] drew its conclusions largely from the established C2C12 myoblast cell line. These cells display a somewhat looser control of the cell cycle (e.g., ref. [57]). Indeed, a later study confirmed that pRb ablation alone induces cell cycle reentry in C2C12, but essentially not in primary myotubes [75].

In primary myotubes, DNA synthesis can be triggered by simultaneously suppressing pRb and the p53 activator ARF. Thus, although the evidence is indirect, it appears that pRb and p53 synergize to prevent cell cycle reentry in primary myotubes. Interestingly, ARF is seemingly deleted in C2C12 cells [75], providing a plausible mechanistic explanation for the lower opposition of these cells to cell cycle reentry [75].

It has also been claimed that concurrent inactivation of pRb and ARF allows TD myocytes (mononuclear, differentiated skeletal muscle cells) to dedifferentiate and proliferate [75]. Unfortunately, this conclusion critically rests on the identification of TD myocytes through the expression of the early differentiation marker, Myogenin. Thus, as it has been shown that Myogenin can be expressed before commitment and is compatible with cell cycle reentry [76], the evidence in favor of the proliferation of former TD myocytes cannot be deemed conclusive.

## 6. Maintenance of the Postmitotic State

It is questionable whether any of the above experimental manipulations, aimed directly at the core cell cycle machinery, allows sustained proliferation of cells derived from myotubes. In fact, it has been described that, in many instances, DNA replication in the reactivated myonuclei—irrespective of their belonging to mono- or multinucleated cells—is incomplete and entails heavy DNA damage [77]. Indeed, it has been proposed that such inability to fully replicate DNA is shared by most TD cells [77]. It has been shown that, in myotubes, incomplete DNA replication is due in part to a defective deoxynucleotide triphosphate (dNTP) pool that limits DNA synthesis. In turn, the deficiency of the dNTP pool is caused by the differentiation-dependent, cell cycle-resistant suppression of genes encoding critical synthetic enzymes, most crucially Thymidine kinase 1 (TK1). However, restoring the dNTP pool allows only partial extension of DNA synthesis, which never reaches completion [77]. 

Many, but not all, cell cycle genes are silenced in myotubes [14] and this is certainly part of the mechanisms preventing the proliferation of TD cells. The di- or trimethylation of histone H3 lysine 27 (H3K27Me2/3) at these genes has been proposed as one important keeper of the postmitotic state. Indeed, many cell cycle genes acquire the repressive H3K27Me2/3 mark and are silenced during skeletal muscle differentiation. At least some of these genes are also repressed in quiescent fibroblasts, but they do not acquire H3K27Me2/3. Thus, this mark is somehow associated with permanent exit from the cell cycle [74]. Importantly, the depletion of pRb in myotubes shows that its continuing presence is required for the maintenance of H3K27Me2/3 at several genes [74] (Figure 3B), adding to the crucial relevance of pRb in the establishment and conservation of the postmitotic state. Interestingly, the Cyclin D1 gene acquires H3K27Me2/3 in myotubes, but in a non-pRb-dependent fashion, probably through the involvement of polycomb group complexes [74].

However, the methylation of H3K27 cannot wholly explain the robustness of the postmitotic state, as most cell cycle genes are readily reexpressed, and presumably lose H3K27Me2/3 [74], following a variety of treatments that reactivate the cell cycle in myotubes [30,40,78].

Altogether, these finding might suggest that TD cells are characterized by obstacles to full DNA replication that lie beyond cell cycle control and pertain to differentiation itself. It is still unclear which changes define the postmitotic state and determine its fundamental attributes.

## 7. Cell Cycle-Unrelated Attack Points

In the 1980s, the then-popular technique of cell fusion was used to show that, when myotubes are fused with proliferating cells to form heterokaryons, their nuclei are driven into S phase [79,80]. The nuclei of many other TD and non-TD cell types could be reactivated in the same way [81], but myotubes were somewhat different: their nuclei could be drawn into S phase by mitogen stimulation only within a few hours of fusion, after which they became refractory to DNA synthesis. In retrospect, these results can probably be explained at the molecular level. In a heterokaryon, nuclei from proliferating cells, when replicating DNA, draw their TD counterparts into S phase through the action of diffusible factors [82], most likely cyclins and cdks. On the other hand, TD muscle nuclei can induce differentiation or inhibit S phase in their non-TD partners by sharing MyoD family proteins [63,64,65]. It should be noted, however, that this explanation is speculative and, to our knowledge, is not supported by direct experiments.

The trisubstituted purine, myoseverin acts on myotube microtubules and induces extensive segmentation into oligo- or mononucleated fragments [83,84]. It has been claimed that such fragments from C2C12 myotubes reenter the cell cycle and proliferate in response to growth factors. However, the methods adopted in these studies analyze muscle cultures as a whole and cannot discriminate between myotube-derived myocytes and contaminating myoblasts. The absence of single-cell analyses severely affects the credibility of the conclusions. Subsequently, independent work failed to reproduce the reported cell cycle reactivation and proliferation effects of myoseverin, although it should be noted that it did not formally disprove them [85].

Myoseverin was also used to induce C2C12 myotube fragmentation, followed by treatment of the “cellulate” thus obtained, according to different protocols. p21 suppression was reported to induce proliferation of the cellulate and transdifferentiation into mesoderm-derived cell types [86]. In a second paper, myoseverin-induced cellulate was treated with disparate small molecules, reportedly triggering transdifferentiation into ectoderm-derived, as well as mesoderm-derived, cells [87]. However, the conclusions of these papers cannot be evaluated, due to serious methodological flaws in the purification and analysis of the myotube fragments.

Some studies attempted to capitalize on knowledge acquired in investigating naturally regenerating organisms. In particular, efforts were focused on the Msx1 gene, which, in the newt, is expressed relatively early in the regenerating blastema [88,89], an undifferentiated tissue that forms in response to amputation in these and other animals. One study by the Keating group [90] claimed that expression of Msx1 in C2C12 myotubes induced dedifferentiation, segmentation into oligo-/mononuclear cells, proliferation, and even redifferentiation into myotubes and other cell types. However, these findings have proven difficult to reproduce and, in fact, have been rejected by at least one study [89].

One year later, the same group reported that an extract from regenerating newt blastema was able to make C2C12 myotubes cleave and proliferate [91]. These results have been scarcely reproduced.

The homeodomain transcription factor Barx2, microinjected into morphologically “immature” primary myotubes, has been reported to induce their cleavage into mononuclear cells, some of which subsequently incorporated BrdU. More “mature” myotubes were resistant to the action of Barx2 and did not cleave [92].

In 2011, Paliwal and Conboy described a method to induce the dedifferentiation and proliferation of myotubes [93]. Their surprisingly simple technique relied on the treatment of myotubes with the tyrosine phosphatase inhibitor BpV(phen) and the apoptosis inhibitor Q-VD-OPh. According to the authors, the latter was not required for dedifferentiation, but merely enhanced the efficiency of the method by preventing myotube death. The work did not attempt to identify the relevant phosphatase(s) and its targets. Strangely, these findings have not been followed up by the authors or, to our knowledge, by any other research group.

Another assault on the postmitotic state exploited the bHLH transcription factor Twist as a probe. Twist is expressed in myoblasts but downregulated upon differentiation. Its forcible expression in C2C12 myotubes initially induced marked downregulation of muscle-specific structural and regulatory genes. This dedifferentiation was accompanied by extensive segmentation and then, with growth factor stimulation, the initiation of DNA synthesis [94]. Mechanistically, it was later found that Twist reduces Myogenin levels, which results in the downregulation of MyoD. In turn, low MyoD levels allow the expression of cyclin D1 and cyclin E2, which promote the transition into S phase [78]. The main results of these two studies have been reproduced in the laboratory of the authors of this review (unpublished data).

## 8. The Apoptosis Connection

The most recent turn in the quest to induce the proliferation of mammalian myotubes establishes a connection between apoptosis and dedifferentiation/proliferation. Using a rigorous methodology, it has been shown that staurosporine-triggered apoptosis induces the fragmentation of mouse myotubes. If apoptosis is subsequently blocked by caspase inhibition before cell death takes place, a small but significant fraction of the mononucleated cells generated through myotube fragmentation reenter the cell cycle and proliferate in vitro. The progeny of the reactivated cells can redifferentiate into myotubes and even contribute to muscle regeneration in vivo [95]. Interestingly, while C2C12 myotube-derived fragments can be made to proliferate simply by inducing and blocking apoptosis as described above, primary myotube fragments require the concurrent knockdown of p53, in agreement with findings already discussed [75].

The link between apoptosis and regeneration is reinforced in a well-established model of amphibian regeneration, newt limb amputation. The authors showed that caspases are activated in the early stages of the response to amputation and remain long active through the entire dedifferentiation phase of the regeneration process, without necessarily causing cell death. Caspase inhibition in the limbs reduced the extent of myofiber dedifferentiation [95].

Collectively, these results strongly indicate that caspases are important players in the dedifferentiation and regeneration processes.

## 9. Concluding Remarks

Inducing proliferation of myotube-derived cells is still an open problem. Remarkably, however, in the last few years, virtually no new reports have been published on this issue, as if it was considered solved. In our view, this is not the case.

### 9.1. Lack of Molecular Understanding

In the first place, none of the available methods to induce myotube proliferation is efficient or readily reproducible. However, even if they were, we would still lack a molecular understanding of what constitutes the postmitotic state. Evidence accumulated in the last sixty years shows that TD cells enter a state of permanent proliferation arrest that is qualitatively different from the stances taken by temporarily or permanently nonproliferating cells (e.g., quiescence and senescence). TD cells do not respond to growth factors with proliferation. If forced into the cell cycle, they suppress their differentiation program. When they reenter S phase, TD cells often face obstacles of unknown nature in completing DNA replication. These features require explanations. Terminal differentiation is an unsolved enigma connected with other complex biological problems, such as regeneration, cancer, cell senescence, and organismal aging. Understanding it would shed considerable light on a vast expanse of biology. Skeletal muscle myotubes are a model system to study terminal differentiation, more amenable than other TD histotypes to experimental investigation. Arguably, the fundamental mechanisms underlying the postmitotic state should be shared by most TD cell types.

### 9.2. Therapeutic Strategies

From a practical standpoint, therapeutic applications are still far into the future. While the skeletal muscle has considerable regenerative capacity, other tissues and organs whose parenchymas are composed of TD cells do not. Examples include the nervous system, sensory organs, the heart (whose cardiomyocyte proliferating capacity is very limited), and endocrine glands. Again, then, the myotube is a model system for TD cell types more difficult to manipulate experimentally. If we succeed in making them proliferate, then very possibly we will be able to do the same with more therapeutically significant target cells. The next challenge will be converting such raw ability into practicable therapeutics, but that is a story yet to be written.
